# Coordination between vapor pressure deficit and CO_2_ on the regulation of photosynthesis and productivity in greenhouse tomato production

**DOI:** 10.1038/s41598-019-45232-w

**Published:** 2019-06-18

**Authors:** Xiao-Cong Jiao, Xiao-Ming Song, Da-Long Zhang, Qing-Jie Du, Jian-Ming Li

**Affiliations:** 1 College of Horticulture, Northwest Agriculture & Forest University, Yangling, 712100 Shaanxi China; 2 College of Horticulture Science and Engineering, Shandong Agricultural University, Taian, 271018 Shandong China

**Keywords:** Biological sciences, Photosynthesis, Stomata

## Abstract

The high vapor pressure deficit (VPD) in some arid and semi-arid climates creates undesirable conditions for the growth of tomato plants (*Solanum lycopersicum* L., cv. Jinpeng). The global CO_2_ concentration ([CO_2_]) has also risen in recent years to levels above 400 μmol·mol^−1^. However, the coordinated effect of VPD and [CO_2_] on tomato plant growth remains unclear, especially at VPDs of 5–6 kPa or even higher that are extremely detrimental to plant growth. Here, we explore the interaction of VPD and [CO_2_] on plant water status, stomatal characteristics, and gas exchange parameters in summer greenhouses in a semi-arid area. Plants were grown in four adjacent glass greenhouses with different environmental conditions: (i) high VPD + low [CO_2_] representing natural/control conditions; (ii) high VPD + high [CO_2_] representing enriched CO_2_; (iii) low VPD + low [CO_2_] representing reduced VPD; and (iv) low VPD + high [CO_2_] representing reduced VPD and enriched CO_2_. Reducing the VPD alleviated the water stress of the plant and increased the gas exchange area of the leaf, which was beneficial to the entry of CO_2_ into the leaf. At this time, the increase of [CO_2_] was more beneficial to promote the photosynthetic rate and then improve the water use efficiency and yield.

## Introduction

Atmospheric drought is a very common phenomenon in arid and semi-arid areas, which usually have a high vapor pressure deficit (VPD) that sometimes exceeds the appropriate level for plant growth and impacts water transport and water balance^1^. Reduced transpiration rate (Tr) at high VPD is observed in most crop species^2–4^. As a result, guard cells may be especially vulnerable to turgor loss under conditions of high evaporation when the low water flux into the stem is insufficient to meet the high Tr. Consequently, stomata close at high VPD, resulting in a decrease in the photosynthetic rate^5^.

A decrease in Tr at high VPD due to the partial closure of the stomata will help in conserving the soil water, but CO_2_ assimilation declines due to the water vapor and CO_2_ exchange synchronization by leaves and canopies^6^. Premature restriction of Tr will result in a decrease in the ability to transport CO_2_ to the leaves^7^, which will have a negative impact on yield^8^.

In conjunction with global warming and climate change, the atmospheric CO_2_ concentration ([CO_2_]) is gradually increasing; levels have risen from about 340 μmol·mol^−1^ in the 1980s to the current level about 410 μmol·mol^−1^, with signs this trend will continue^9^. CO_2_ diffuses through stomata into the intercellular spaces of leaves and then spreads through the mesophyll to carboxylation sites in the chloroplasts^10^. Elevated [CO_2_] can overcome its diffusion limitations^11,12^, reduce photorespiration^13^, enhance RuBp regeneration^14^, and promote efficient assimilation. On the other hand, elevated [CO_2_] can compensate for the effects of drought on water status^15^. In C3 plants, rising [CO_2_] reduces the sensitivity of assimilation rates caused by high VPD to partial stomatal closure^16^.

Stomata regulate the CO_2_ uptake and water loss of leaves^17^. The stomatal responses to VPD are actively driven by the phytohormone abscisic acid (ABA)^18^ and regulated by rapid gene expression^19^. Stomatal morphology parameters are also significantly affected by VPD^20^. Active physiological stomatal control in response to [CO_2_] will eventually initiate ion pumping to alter the turgor pressure of guard cells^21^. These mechanisms are closely associated with the capacity to sustain stomatal conductance (gs) and to optimize photosynthesis. VPD, [CO_2_], and their interaction affect plant transpiration and assimilation^22^. Angiosperm stomata usually exhibit short-term rapid responses; for example, light irradiance promotes openness but increased [CO_2_] and VPD promote closure^23^. The sensitivity of stomatal conductance to VPD is reduced at elevated [CO_2_]^24^.

Our research has shown that controlling VPD can benefit protected cultivation systems^20,25–27^, similar to other findings^28,29^. However, most such studies focus on VPD levels below 4 kPa and few consider VPD up to or even beyond 5–6 kPa^30^, yet this represents the climate characteristics of most arid and semi-arid areas, such as northwest China. The highest VPD in a summer greenhouse in this region can be up to 6 kPa, but a suitable VPD for tomato growth is less than 2 kPa^31^. Little research has been done on reducing VPD while increasing [CO_2_] under these circumstances.

We hypothesized that: (1) lowering the VPD would effectively alleviate the water stress of the leaves, prevent the excessive transpiration that leads to stomatal closure, and thus maintain the water balance of plants and (2) under low VPD conditions, the morphology or density of the stomata may change, increasing the gas exchange area between the interior of the leaves and the outside environment to better balance water loss and CO_2_ absorption so CO_2_ can quickly enter the leaves and promote photosynthesis. We tested this hypothesis in a greenhouse study of tomato plants (*Solanum lycopersicum* L., cv. Jinpeng) considering four different environmental conditions: (i) high VPD + low [CO_2_] representing natural/control conditions (HVPD-LCO_2_); (ii) high VPD + high [CO_2_] representing enriched CO_2_ (HVPD-HCO_2_); (iii) low VPD + low [CO_2_] representing reduced VPD (LVPD-LCO_2_); and (iv) low VPD + high [CO_2_] representing reduced VPD and enriched CO_2_ (LVPD-HCO_2_). The transpiration rates of the plants exposed to low- and high-VPD were compared. The water status of the leaves was also determined to verify whether excessive transpiration was avoided and the whether stomatal openings were prevented from fully or partially closing. The ability of the plant to photosynthesize was measured to improve the understanding of how the VPD and CO_2_ have a coordinated effect on plant growth as well as what conditions are more conducive for CO_2_ to enter the leaves. Accordingly, the biomass, yield, and water use efficiency were measured and compared.

## Results

### Environmental data

**Figure 1:**
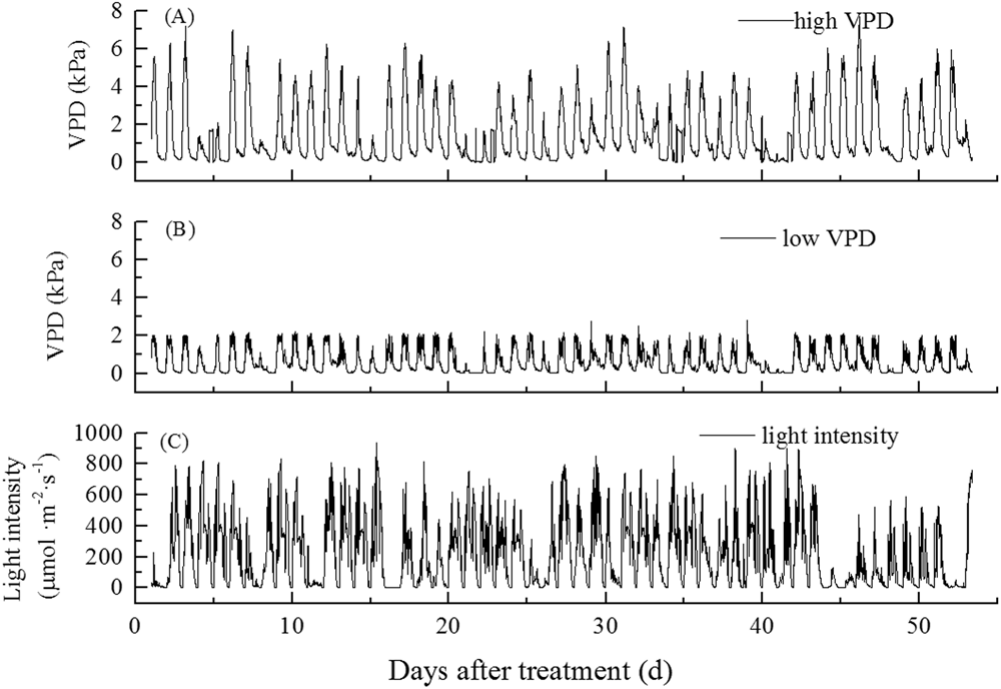
Daily changes of VPD (from 8:00 to 20:00) in control (high) (**A**) and low VPD conditions (**B**), and light intensity (**C**) during the experiment period.

The experimental design successfully achieved very different VPD values in the high- and low-VPD greenhouses. Based on recording VPD changes from 8:00 to 20:00 h every day, the mean of the highest daily VPD values was 4.44 kPa for high VPD conditions and 1.90 kPa for low VPD conditions (Fig. 1). The VPD exceeded 4, 5, 6, and 7 kPa for 36, 20, 10, and 3 days, respectively. Greenhouses with low VPD conditions had levels maintained below 2 kPa.

### Coordination between VPD and CO_2_ on leaf stomatal traits after long-term acclimation

Reducing the VPD under ambient and elevated [CO_2_] conditions significantly increased the stomatal density (SD) by 24.80 and 42.90%, respectively. Stomatal length and width respectively increased by 12.40 and 13.20% in plants exposed to the LVPD + LCO_2_ environment and by 6.0 and 4.8% in plants exposed to the LVPD + HCO_2_ environment compared to the control. No significant differences in stomatal characteristics were observed between plants exposed to high- and low-[CO_2_] environments under high VPD conditions (Table 1).

**Table 1:** Coordination between VPD and CO_2_ on stomatal characteristics.

Treatment	Stomatal density (mm^−^^2^)	Stomatal length (μm)	Stomatal width (μm)
HVPD-LCO_2_	167.2 ± 3.8 c	31.5 ± 0.3 c	22.7 ± 0.2 c
HVPD-HCO_2_	174.9 ± 5.4 c	31.7 ± 0.3 c	22.9 ± 0.2 c
LVPD-LCO_2_	208.7 ± 3.7 b	35.4 ± 0.4 a	25.7 ± 0.2 a
LVPD-HCO_2_	239.0 ± 5.3 a	33.4 ± 0.3 b	23.8 ± 0.2 b
VPD	**	**	**
CO_2_	**	**	**
VPD × CO_2_	*	**	**

Values are means ± SE (n = 40). Significant differences between the four treatments were examined using Tukey’s test. Different small letters in a column indicate significant differences among treatments at the 0.05 significance level. *Significant at P < 0.05, indicates the difference was significant; **Significant at P < 0.01, indicates the test factor has an extremely significant effect on the indicator; ns: non-significant difference.

### Coordination between VPD and CO_2_ on plant water status

**Figure 2:**
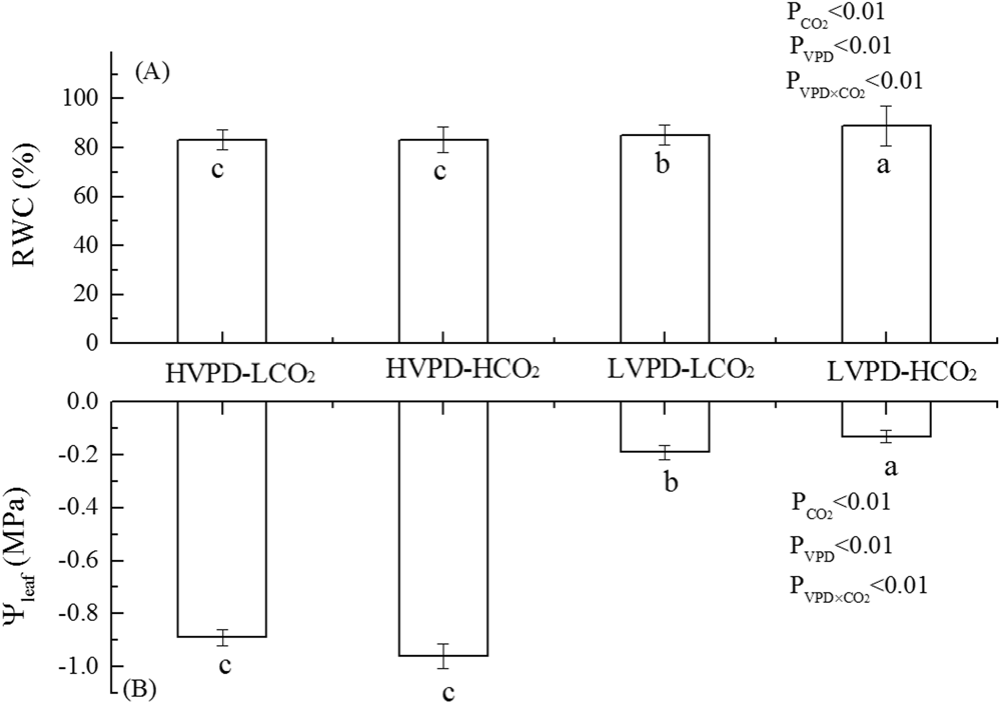
Coordination between VPD and CO_2_ on leaf water content (**A**) and leaf water potential (**B**).

Regulation of VPD and [CO_2_] strongly affected plant water status. Reducing VPD significantly increased the relative water content (RWC) and water potential (ψ_leaf_) of plant leaves (Fig. 2). Reduced VPD at atmospheric [CO_2_] resulted in increases in the RWC and ψ_leaf_ by 2.33 and 78.65%, respectively; corresponding increments in the high-[CO_2_] environment were 6.89 and 85.39%. The leaves maintained a higher water status under low VPD conditions, which may be beneficial for CO_2_ entrance.

### Coordination between VPD and CO_2_ on leaf gas exchange

**Figure 3:**
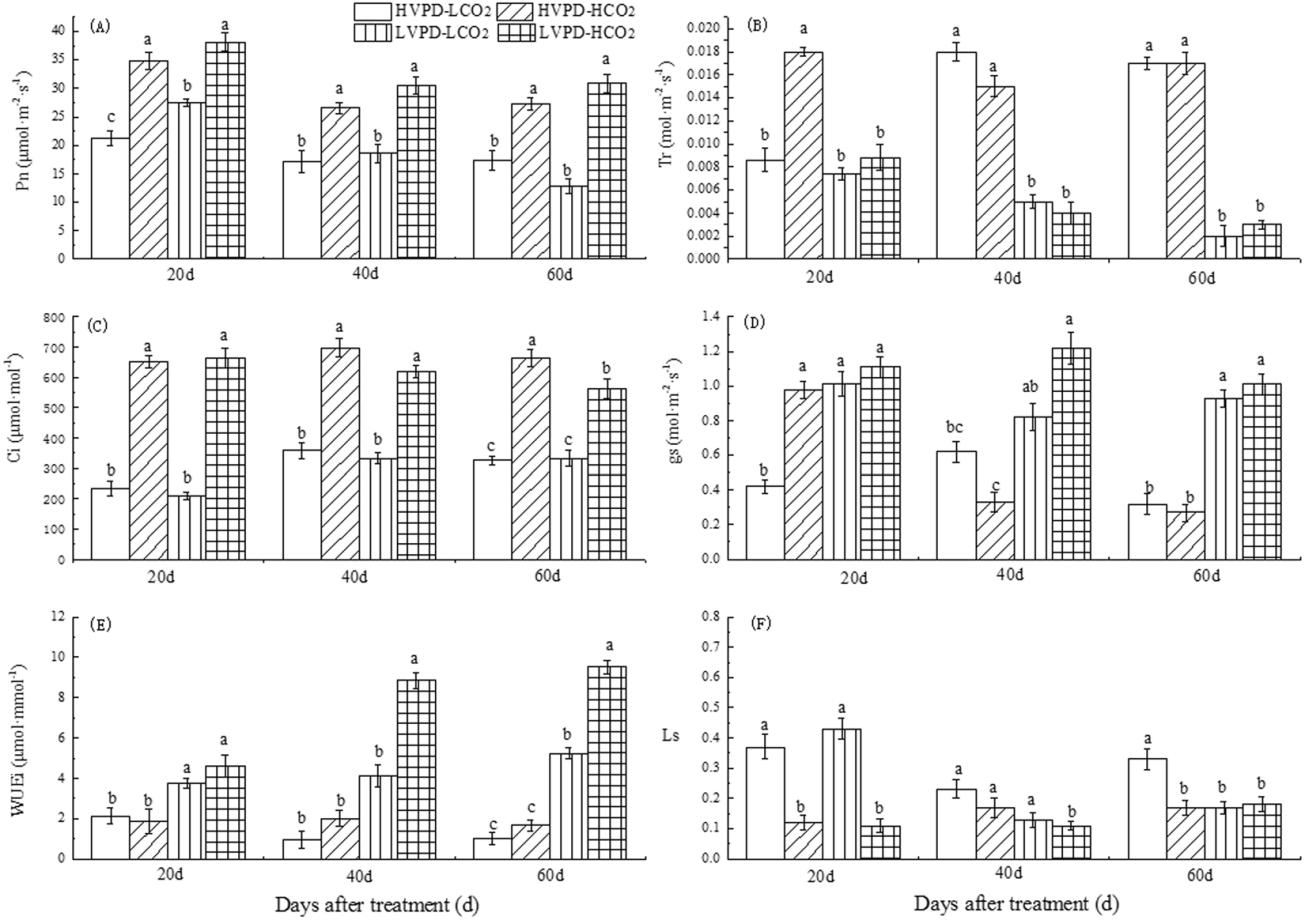
Coordination between VPD and CO_2_ on leaf gas exchange parameters.

The intercellular CO_2_ concentration (Ci) significantly increased in plants exposed to HVPD + HCO_2_ or LVPD + HCO_2_ conditions (Fig. 3). The photosynthesis rate (Pn) significantly increased by 63.52, 55.11, and 57.64% on day 20, 40, and 60 for plants exposed to the HVPD + HCO_2_ environment, respectively; by 79.27, 77.88, and 78.41% on day 20, 40, and 60 for plants exposed to the LVPD + HCO_2_ environment, respectively; and by 29.10% on day 20 for the LVPD + LCO_2_ environment. The gs increased significantly by 32.26 and 190.63% for plants exposed to LVPD + LCO_2_ conditions and by 96.77 and 215.63% for plants exposed to LVPD + HCO_2_ conditions on day 40 and 60, respectively. Corresponding decreases in Tr were 72.22 and 88.24% for plants exposed to LVPD + LCO_2_ conditions and 77.78 and 82.35% for plants exposed to LVPD + HCO_2_ conditions. As a result, the RWC and ψ_leaf_ for plants exposed to LVPD + LCO_2_ conditions and LVPD + HCO_2_ conditions both increased significantly (Fig. 2). The instantaneous water use efficiency (WUEi) significantly increased by 74.77 and 412.75% at 20 and 60 days for plants exposed to LVPD + LCO_2_ conditions and by 114.49, 811.34, and 833.33% at 20, 40, and 60 days for plants exposed to LVPD + HCO_2_ conditions (Fig. 3).

### Coordination between VPD and CO_2_ on CO_2_ response curves and light response curves

Determination of photosynthetic physio-ecological characteristics of plants through CO_2_ and light response curves has become a hot issue in scientific research^32^. The Pmax-CO_2_ (maximum photosynthesis rate on the CO_2_ response curve) increased by 10.12 and 35.20% in plants exposed to HVPD + HCO_2_ and LVPD + HCO_2_ environments, respectively, but decreased by 2.08% in plants exposed to the LVPD + LCO_2_ environment compared to the control. The interval between the CO_2_ compensation point (CCP) and CO_2_ saturation point (CSP) was used to characterize the range of [CO_2_] that plants could use for net photosynthesis^33^. The interval was 900.64 μmol·m^−2^·s^−1^ for plants exposed to HVPD + LCO_2_ conditions, 785.17 μmol·m^−2^·s^−1^ for HVPD + HCO_2_ conditions, 797.27 μmol·m^−2^·s^−1^ for LVPD + LCO_2_ conditions, and 921.52 μmol·m^−2^·s^−1^ for LVPD + HCO_2_ conditions. This result shows elevated [CO_2_] can decrease the interval at high VPD conditions but increases the interval under low VPD conditions. The interval for LVPD + HCO_2_ conditions was the largest, indicating that plants had the strongest adaptability to different [CO_2_] for photosynthesis under these conditions.

**Figure 4:**
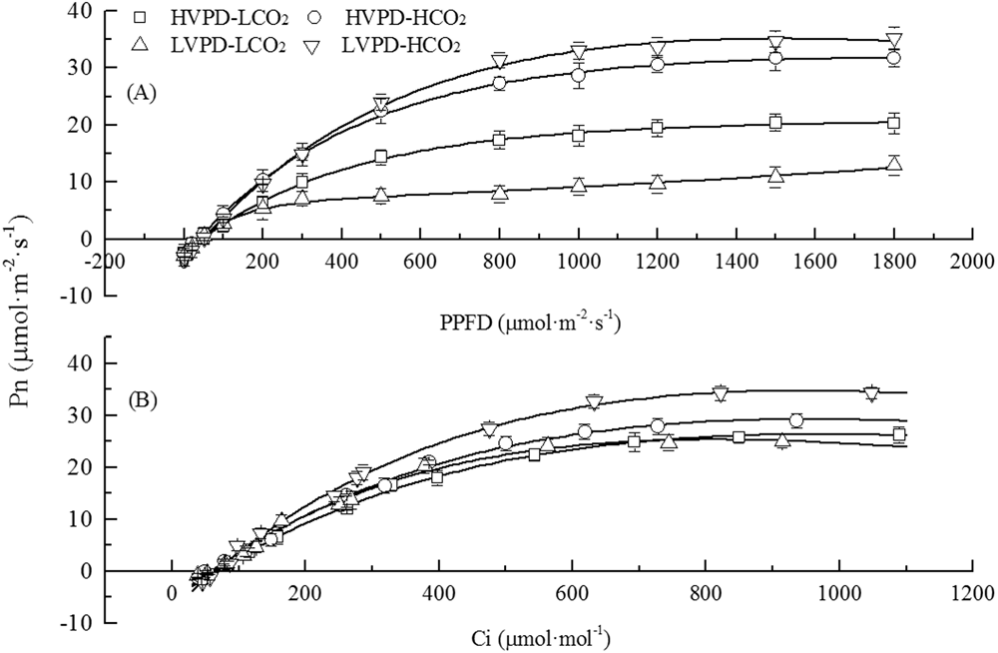
Responses of CO_2_ assimilation rate to photosynthetic light intensity (PPFD) (**A**) and intercellular CO_2_ concentration (Ci) (**B**) in leaves of tomato plants.

The order of Pmax-light (maximum photosynthesis rate in light response curve) of plants among treatments was LVPD + HCO_2_ > HVPD + HCO_2_ > HVPD + LCO_2_ > LVPD + LCO_2_ (Fig. 4). The Pmax-light of plants exposed to HVPD + HCO_2_ and LVPD + HCO_2_ conditions significantly increased by 56.16 and 73.92%, respectively, compared to the control. The interval between the light compensation point (LCP) and light saturation point (LSP) indicated the range of light that plants could use for net photosynthesis. This interval was 1622.34 μmol·m^−2^·s^−1^ for plants exposed to the HVPD + LCO_2_ environment, 1720.33 μmol·m^−2^·s^−1^ for the HVPD + HCO_2_ environment, 1438.23 μmol·m^−2^·s^−1^ for the LVPD + LCO_2_ environment, and 1868.25 μmol·m^−2^·s^−1^ for the LVPD + HCO_2_ environment. The interval for the LVPD + HCO_2_ environment was the largest, indicating that plants had the strongest adaptability to different light intensities for net photosynthesis in these conditions. The dark respiration (Rd) of plants exposed to HVPD + LCO_2_, HVPD + HCO_2_, LVPD + LCO_2_ and LVPD + HCO_2_ was 2.69, 2.83, 1.18 and 3.73 μmol·m^−2^·s^−1^.

### Coordination between VPD and CO_2_ on plant growth

**Figure 5:**
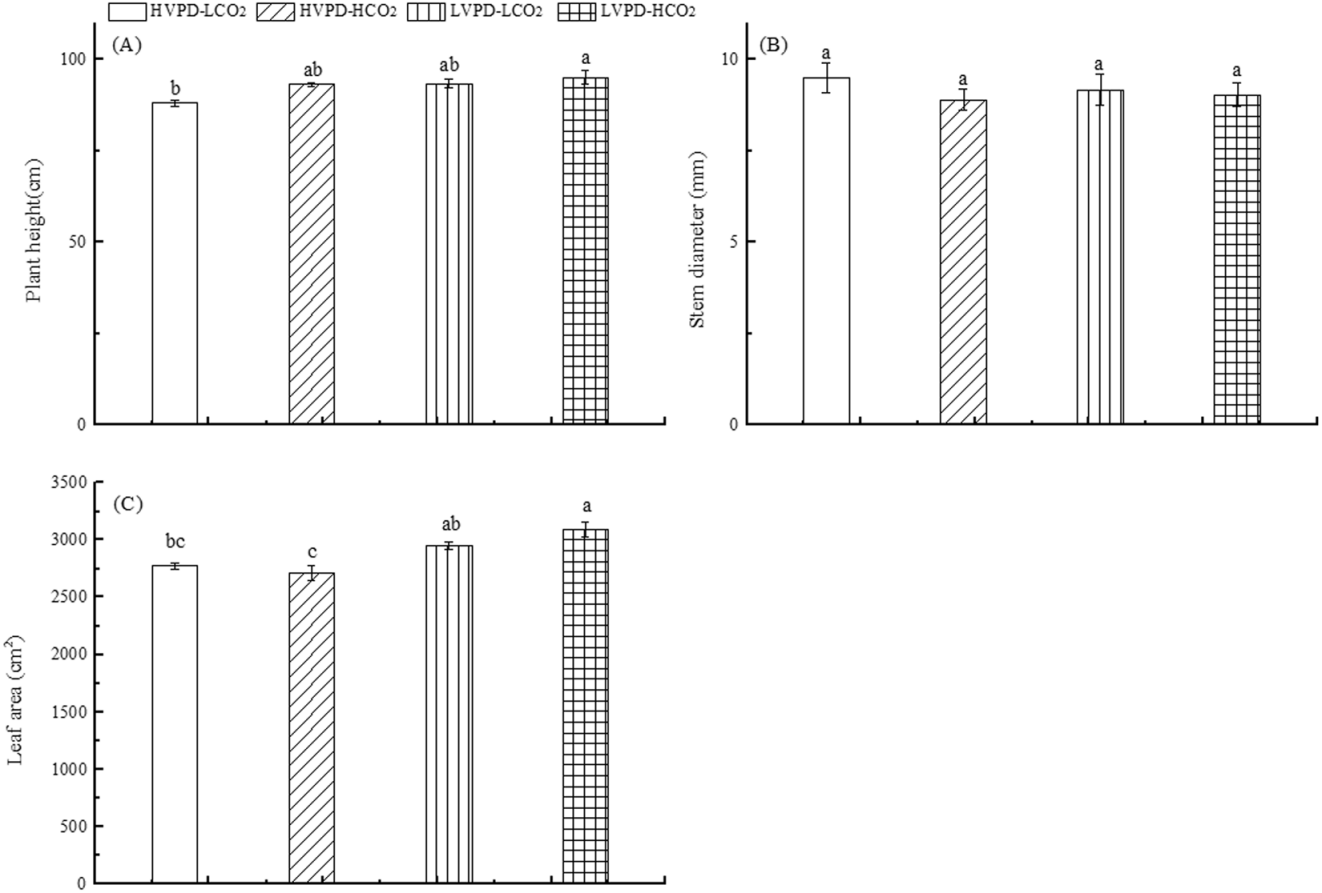
Coordination between VPD and CO_2_ on plant height (**A**), stem diameter (**B**), and leaf area (**C**).

Changes in plant morphology directly reflect adaptability to the growing environment^34^. Plant stem diameter did not significantly differ under the four treatments considered. The average height of plants exposed to HVPD + LCO_2_, HVPD + HCO_2_, LVPD + LCO_2_, and LVPD + HCO_2_ environments was 88.2, 93.25, 93.60, and 95.20 cm, respectively. Among these values, plant height in the LVPD + HCO_2_ conditions was significantly greater than the control (Fig. 5).

**Figure 6:**
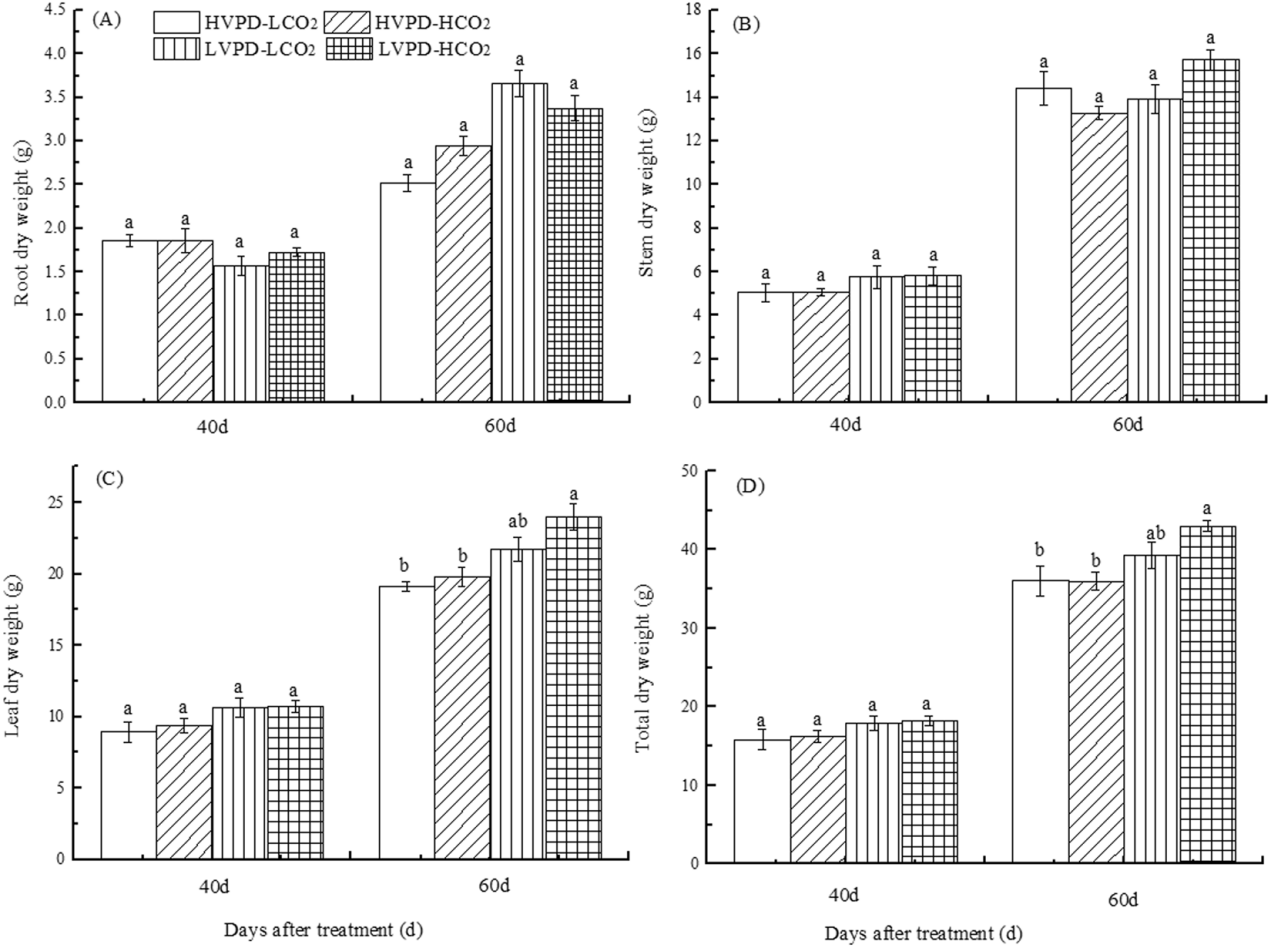
Coordination between VPD and CO_2_ on plant biomass allocation.

Leaves are the largest part of the plant to contact the external environment, and changes in environmental factors directly affect their traits and functions. Leaves play an important role in the relationships between plant water status, light energy interception, and energy balance^35,36^. The leaf area of plants exposed to HVPD + HCO_2_ conditions was 2.06% smaller than the control, but for LVPD + LCO_2_ and LVPD + HCO_2_ conditions was respectively 6.35 and 11.53% larger than the control (Fig. 5). With growth of the plant, the proportion of root dry weight decreased gradually, of stem dry weight increased gradually, and of leaf dry weight remained unchanged, with no significant differences noted in these values. The leaf and total dry weight of plants exposed to LVPD + LCO_2_ and LVPD + HCO_2_ conditions were both greater than the control, with only the latter comparison being significant (Fig. 6). These findings indicate that reducing VPD can increase plant biomass mainly by increasing leaf biomass. Reducing VPD while elevating [CO_2_] can significantly promote biomass accumulation.

### Coordination between VPD and CO_2_ on water consumption and yield

**Figure 7:**
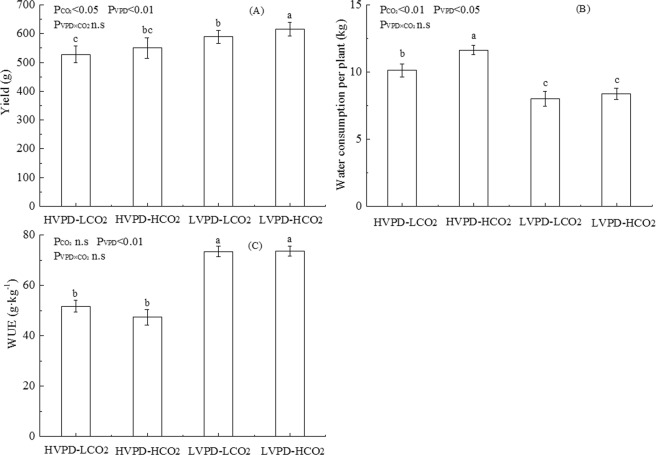
Coordination between VPD and CO_2_ on plant yield (**A**), water consumption per plant (**B**), and WUE (**C**).

The average water consumption per plant for plants exposed to HVPD + HCO_2_ conditions significantly increased (by 14.91%) and for plants exposed to LVPD + LCO_2_ and LVPD + HCO_2_ significantly decreased (by 20.83 and 17.18%, respectively) compared to the control (Fig. 7). Water saved per plant for plants exposed to LVPD + LCO_2_ and LVPD + HCO_2_ conditions was 2.01 and 1.74 kg, respectively, compared to the control. Average yield per plant for plants exposed to LVPD + LCO_2_ and LVPD + HCO_2_ conditions increased significantly (by 11.53 and 16.75%, respectively). Two-factor analysis showed that [CO_2_] and VPD both had significant effects on plant water consumption and yield, both of which increased under HVPD + HCO_2_ conditions. Reducing the VPD decreased plant water consumption but increased plant yield and water use efficiency (WUE). The VPD also had a significant effect on WUE.

## Discussion

### Reduced VPD kept leaves in a higher water state and improved plant water use efficiency

VPD is the driving force for water flow in the soil-plant-atmosphere continuum. Increasing VPD leads to a high evaporation demand. The balance between water supply and evaporative demand is lost under high VPD, causing leaf dehydration and water stress (i.e., leaf wilting, xylem cavitation)^37^. In this experiment, the low VPD conditions in the greenhouse were effectively maintained below 2 kPa, which is suitable for tomato plant growth. Plants exposed to low VPD conditions did not show excessive transpiration, because of the reduced transpiration pull and the leaves maintained a higher water content and water potential. As such, the changes in Tr were consistent with the changes in RWC and ψ_leaf_. The significant increase in the length and width of the stomata were observed. Together, these indicators indicate that no significant stomatal closure has occurred.

Two-factor analysis showed that VPD, [CO_2_] and their interactions had significant effects on RWC and leaf water potential. The RWC of plants exposed to LVPD + HCO_2_ was greater than for plants exposed to LVPD + LCO_2_. A possible reason is that the greater [CO_2_] provides more material for plant photosynthesis; the resulting increase in organic matter decreases the osmotic potential of guard cell and increases the amount of water absorbed^38^.

Both the WUEi and WUE of plants exposed to low VPD conditions increased significantly, indicating low VPD conditions are conducive to water use efficiency. Among them, the increase of WUEi of LVPD + HCO_2_ is larger, indicating that the application of CO_2_ under low VPD is more conducive to the instantaneous utilization efficiency of water. The improvement in the WUE of plants exposed to low VPD conditions was due to the increase in yield and decrease in water consumption during plant growth. When irrigation water is scarce, it is feasible to reduce VPD by adding atomized water to the air^39^. Of course, the water consumption of the atomization system should also be evaluated. The WUEi increase in plants exposed to high [CO_2_] conditions is consistent with previous research^40,41^, indicating increased [CO_2_] can confer drought resistance by reducing carbon starvation^42^. This is also an important aspect of research under drought conditions.

### Coordination of VPD and CO_2_ stimulated plant growth by enhancing photosynthetic capacity

Arve *et al*.^43^ found that, during the light period, stomatal aperture was 25% smaller under low relative humidity (RH) than continuously high RH. In the present experiment, stomatal density, length, and width significantly increased in plants exposed to LVPD + LCO_2_ or LVPD + HCO_2_ conditions compared to the control. The gs of plants exposed to HVPD + LCO_2_ conditions was significantly smaller than those under low VPD conditions after both 40 and 60 days. Similarly, *Ramonda nathliat*e maintained small mesophyll cells and lower gs^44^ under severe drought conditions to increase the ability to resist drought^45^. This also indicates that reducing the VPD increases the gas exchange area between the leaves and outside environment. For plants exposed to LVPD + HCO_2_ conditions, the stomatal limitations (Ls) decreased and gs increased (Fig. 3), which were helpful for CO_2_ entrance into stomata. The Pn of plants exposed at HVPD + HCO_2_ and LVPD + HCO_2_ treatment were both increased significantly, indicating that high [CO_2_] is beneficial to the photosynthesis of plants, and the larger increase of the latter indicates that low VPD conditions were more conducive to plant CO_2_ utilization. It was also verified that low VPD environment is more conducive to CO_2_ entering into stomata.

High [CO_2_] is favorable for Pn improvement for a short time, but photosynthetic acclimation usually occurs after long-term treatment^46,47^. However, in the present experiment the Pn of plants exposed to HVPD + HCO_2_ and LVPD + HCO_2_ conditions was significantly larger than the control at every stage of plant growth, with no evidence of photosynthetic acclimation. In the former condition, this may be due to the decrease of sensitivity of [CO_2_] in the late growth stage; in the latter condition, it may be due to the lack of reduction in the demand for CO_2_ in the later stages of growth.

The Rubisco activation usually has a decrease at high VPD^46^. In present study, VPD was reduced below 2 kPa at low VPD conditions, the stress of air on plants is alleviated, which can effectively avoid the inactivation of Rubisco. The intervals between CCP and CSP and between LCP and LSP were the largest for plants exposed to LVPD + HCO_2_ conditions, indicating these plants had the strongest adaptability to the widest [CO_2_] range and widest light intensity range for net assimilation. Furthermore, elevated [CO_2_] can increase Pn by inhibiting photorespiration^48^. The Rd was the highest in plants exposed to LVPD + HCO_2_ conditions, accompanied by the maximum photosynthetic rate. Similar to previous studies, there was a positive relationship between CO_2_ assimilation rate and dark respiration rate, that meeting the energy demand for plant growth simultaneously^49^.

## Conclusion

In the present study, the LVPD + HCO_2_ environment affected plant growth in two aspects. The first effect is related to the water relationship. The water potential and turgor pressure of leaves in low VPD conditions increased due to the reduced transpiration pull. When the products of photosynthesis increase, the osmotic potential of the leaf decreases, the guards cells absorb more water, and the leaves maintain a higher moisture state, which can keep the stomata open^50^. This is confirmed by the significant increase in stomata size under low VPD conditions along with the increase in both stomatal density and leaf area. These factors all lead to the increase of gas exchange area between the leaves and the external environment. The second is related to the photosynthetic capacity. The increase in gas exchange area is conducive to CO_2_ entering the leaves and providing more raw material for photosynthesis. At the same time, higher [CO_2_] can prevent RuBp oxidation and reduce CO_2_ loss in photorespiration. The CO_2_ and light response curves indicate the plants exposed to the LVPD + HCO_2_ environment had the highest utilization ability for CO_2_ and light and the strongest ability to photosynthesize. The findings of this research are particularly significant with respect to how plants in arid and semi-arid regions will respond to future increases in atmospheric [CO_2_].

## Materials and Methods

### Experimental conditions and plant materials

The experiment was carried out from April 1 to June 21, 2017 at Yangling, located in Shannxi province in northwestern China (N 34°15´, E 108°04´, 443.6 m above sea level) in a semi-arid climate zone. Tomato seeds *(Solanum lycopersicum* L. cv. ‘Jinpeng’) were germinated in an artificial climate chamber with day/night temperatures of 25 °C/18 °C, a 12 h light (400 μmol·m^−2^·s^−1^)/12 h dark photoperiod, and RH of 80%. Seedlings were planted in cultivation substrate when plants had four leaves and a center leaf, with pots then placed in four independent but closely connected glass greenhouses, each 3 m wide and 6 m long, for a floor area of 18 m^2^; this was considered day 0. At midday on a sunny day, the light intensity in the greenhouses reached 800–900 μmol·m^−2^·s^−1^ (Fig. 1C). Plant density was 3 plants/m^2 ^^25^. A total of 30 plants per treatment were randomly sampled at each time point, with at least three repeated measurements taken for each indicator. For each plant, two trusses were used with four fruits per truss. The substrate surface was covered with a black plastic film to prevent water loss due to evaporation and keep the substrate water content at 85–90% of field water capacity. The amount of irrigation water/water consumption equaled the amount of plant transpiration. Pots were weighed at 7 d intervals with an electronic scale. The RH, temperature (T), and light intensity were recorded automatically every 5 minutes using sensors (ZDR-20j, WuGe Instruments Co., Ltd., China). VPD values were calculated using^51^

1 $${\rm{VPD}}={\rm{0}}{{\rm{.611e}}}^{(\mathrm{17}{\rm{.502}}\ast {\rm{T}}/({\rm{T}}+{\rm{240}}\mathrm{.97}))}\ast ({\rm{1}}\,-\,{\rm{RH}}){\rm{.}}$$

Other management considerations were the same as field management.

### Environmental design

The two independent variables in this experiment—VPD and [CO_2_]—were each considered at two levels, for a total of four treatments: natural conditions of high VPD and low [CO_2_] (HVPD-LCO_2_) as the control; high VPD with high [CO_2_] (HVPD-HCO_2_) representing enriched CO_2_; low VPD with low [CO_2_] (LVPD-LCO_2_) representing reduced VPD; and low VPD with high [CO_2_] (LVPD-HCO_2_) representing reduced VPD and enriched CO_2_. High natural VPD varied with the weather, with the maximum value exceeding 6 kPa. Low VPD was controlled under 2 kPa by a high-pressure industrial humidifier (model BL-C03ZT; BELIN, Shanghai, China); the machine started automatically when the VPD in the greenhouse exceeded 2 kPa. High [CO_2_] was about 800 ± 50 μmol·mol^−1^, supplied by a liquefied CO_2_ cylinder. Natural low [CO_2_] was about 400 ± 50 μmol·mol^−1^. VPD and [CO_2_] were not adjusted on rainy days.

### Measurement of leaf water status

Leaves were weighed to determine the leaf fresh weight (Wf), then soaked in distilled water for about 24 hours until they were saturated and weighed again (Wt), then dried at 80 °C to a constant weight (Wd). The RWC was calculated as follows^52^:

2 $${\rm{RWC}}=({\rm{Wf}}\,-\,{\rm{Wd}})/({\rm{Wt}}\,-\,{\rm{Wd}})\times \mathrm{100} \% {\rm{.}}$$

Leaf water potential (ψ_leaf_) was measured between 12:00 and 13:00 h using a pressure chamber (PMS-1000, Corvallis, OR, USA)^53^.

### Measurement of gas exchange parameters

Leaf gas exchange parameters of plants were measured on new fully expanded leaves on sunny days between 9:00 and 11:00 h, using a portable photosynthesis system (LI-6800, Li-Cor, Inc., Lincoln, NE, USA). The environmental parameters of the cuvette were set close to the external environment in the greenhouse during measurement: high VPD conditions were set at 35 °C/30%; low VPD conditions were set at 35 °C/70%. If the VPD is set directly, the range of T or RH may exceed the range of suitable tomato growth even if they meet the VPD setting values. For this reason, we set T and RH directly to conditions suitable for tomato growth instead of setting the VPD directly. Light intensity was set at 1000 µmol·m^−2^·s^−1^; high and low [CO_2_] were set at 800 ± 10 and 400 ± 10 µmol·mol^−1^, respectively; flow rate was set at 500 µmol·s^−1^; fan speed was set at 10000 rpm; and the red:blue ratio was set at 9:1. The stomatal limitation (Ls) was calculated according to^54^:

3 $${\rm{Ls}}={\rm{1}}\,-\,{\rm{Ci}}/\mathrm{Ca},$$

where Ci is the intercellular [CO_2_] and Ca is the atmospheric [CO_2_].

### Measurement of CO_2_ response curves and light response curves

The CO_2_ and light response curves were measured on the leaves by determining gas exchange parameters. For the CO_2_ response curves, the CO_2_ gradient was set at 400, 300, 200, 150, 100, 50, 30, 400, 400, 600, 800, 1000, and 1200 μmol·mol^−1^, with a light intensity of 1000 μmol·m^−2^·s^−1^. For the light response curves, the light intensity gradient was set at 1800, 1500, 1200, 1000, 800, 400, 300, 200, 100, 50, 20, and 0 μmol·m^−2^·s^−1^, with high and low [CO_2_] of 800 ± 10 and 400 ± 10 µmol·mol^−1^, respectively.

### Measurement of stomatal characteristics

Stomatal characteristics were measured on the same leaves used for measurement of gas exchange parameters. A 1-cm^2^ patch of clear nail polish on the left or right side of the main vein on the abaxial surface of leaves was used to make a transparent imprint. The nail polish was removed with tweezers after it dried and then placed on a glass slide. These leaf peels were then examined using a microscopic imaging system (BX51 + IX71, Olympus, Japan) at both 200× and 400× magnification. For each leaf peel, we selected and photographed 40 views at random at 200× magnification to determine the stomatal density (SD). Measurements of stomatal length and width were derived from photographs taken at 400× magnification^55^. Images were analyzed using the public domain image processing program ImageJ (ImageJ, U.S. National Institutes of Health, Bethesda, Maryland, USA).

### Measurement of plant growth, yield, and WUE

Plant height was measured from the surface of the cultivation substrate to the top of the plant. Stem diameter was measured with a Vernier caliper at 1 cm above the substrate surface. Leaf area was measured on day 0, 30, and 50 using a leaf area meter (LI-COR, Inc., Lincoln, Nebraska, USA). Biomass was measured on day 40 and 60.

Instantaneous water use efficiency (WUEi) was measured by a portable photosynthesis system (LI-6800, Li-Cor, Inc., Lincoln, NE, USA) and calculated as follows^56^:

4 $${\rm{WUEi}}={\rm{Pn}}/{\rm{Tr}}$$

The fruit was harvested after the color-turning period and counted to determine yield. WUE was determined from plant yield. Water consumption was calculated from the following equation:

5 $${\rm{WUE}}={\rm{yield}}/{\rm{water}}\,{\rm{consumption}}{\rm{.}}$$

### Statistical analysis

Data were analyzed with SPSS (Version 16.0; IBM Institute, USA) using one-way and two-way analysis of variance (ANOVA) to evaluate the effects of VPD, [CO_2_], and their interaction on all parameters. Differences between treatments were compared using Tukey’s test. Differences were considered significant at P < 0.05.

## Data Availability

The datasets generated and/or analyzed during the current study are not publicly available, but can be requested from the corresponding author.
